# RIDDLE: reflective diffusion and local extension reveal functional associations for unannotated gene sets via proximity in a gene network

**DOI:** 10.1186/gb-2012-13-12-r125

**Published:** 2012-12-26

**Authors:** Peggy I Wang, Sohyun Hwang, Rodney P Kincaid, Christopher S Sullivan, Insuk Lee, Edward M Marcotte

**Affiliations:** 1 Department of Biomedical Engineering, The University of Texas at Austin, 2500 Speedway, Austin, TX 78712, USA; 2 Center for Systems and Synthetic Biology, Institute for Cellular and Molecular Biology, University of Texas at Austin, 2500 Speedway, Austin, TX 78712, USA; 3 Department of Biotechnology, College of Life Science and Biotechnology, Yonsei University, 50 Yonsei-ro, Seodaemun-gu, Seoul, 120-749, Korea; 4 Molecular Genetics and Microbiology, College of Natural Sciences, University of Texas at Austin, 2506 Speedway, Austin, TX 78712, USA; 5 Department of Chemistry and Biochemistry, University of Texas at Austin, 2500 Speedway, Austin, TX 78712, USA

## Abstract

The growing availability of large-scale functional networks has promoted the development of many successful techniques for predicting functions of genes. Here we extend these network-based principles and techniques to functionally characterize whole sets of genes. We present RIDDLE (Reflective Diffusion and Local Extension), which uses well developed guilt-by-association principles upon a human gene network to identify associations of gene sets. RIDDLE is particularly adept at characterizing sets with no annotations, a major challenge where most traditional set analyses fail. Notably, RIDDLE found microRNA-450a to be strongly implicated in ocular diseases and development. A web application is available at http://www.functionalnet.org/RIDDLE.

## Background

In the modern age of high-throughput genetic studies, functional enrichment analyses remain a vital approach to analyzing data. Microarray, mass spectrometry, genome-wide association, and other genome-level studies commonly produce query gene sets - gene sets of interest, often containing many uncharacterized members, from which coherent biological modules need to be identified. There exist many methods that attempt to discover known biological functions involved with a query set, most of which fall under one of three broad categories: overlap-based, rank-based, and local network-based.

**Figure 1:**
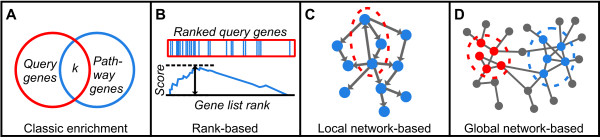
A summary of four basic types of tests identifying functional pathway enrichment of a query gene set. **(a) **In the classic enrichment test, the significance of genes shared by the query and pathway set is assessed. **(b) **In a rank-based test, each gene is first ranked by an appropriate score, for example, differential expression. A pathway is significant if the corresponding genes are located near the top or bottom of the list. **(c) **Local network-based methods consider the known topological information between pathway genes. **(d) **Global network-based methods consider a genome-scale map of known genetic associations.

In the classic overlap-based enrichment analysis (Figure 1a), the functional annotations for the genes in the query set are examined. An annotation is enriched if it is present in the gene set at a greater than expected frequency, the significance of which may be computed through a statistical test (for example, the hypergeometric test [1]). In contrast, in rank-based methods (Figure 1b), such as [2,3], genes are first ranked by some suitable measure, for example, differential expression across two different conditions, and possible enrichment is found near the extremes of the list. Rank-based methods are usually highly specialized for gene expression array analysis. Both overlap and rank-based methods require the queried genes to be sufficiently annotated.

In some more recently developed local-network methods (Figure 1c), a query set is compared against the genes and internal interactions of a known functional pathway. These interactions may be visualized as a map or network, in which nodes represent genes and connecting edges represent interactions. While these methods move towards the idea of finding contributive information from gene networks, they often require sophisticated information about a single pathway under investigation, such as a detailed sub-network of interactions [4-6], directionality of edges [7-9], or additional interactions between shared and non-shared genes of the query and pathway sets [10].

In principle, substantial benefit can be achieved by considering a global network of gene or protein interactions. Many such networks have become available in recent years (for example, [11-17]), compiled from various independent lines of evidence into a rich resource fit for facilitating systematic functional analyses. In these networks, interacting, co-expressed, and other evidently associated genes are linked to or lie close to one another in the topology, facilitating the application of guilt-by-association (GBA) methods for predicting functionally associated genes. Indeed, GBA principles have allowed accurate predictions of not only functionally associated genes, but also genes underlying phenotypes and diseases [12,16-24]. The concept of utilizing both direct and indirect linkages between genes has been widely explored (for example, [22,25,26]). In particular, diffusion algorithms, which spread information across the network topology, have been extensively studied and shown to be extremely effective across numerous settings (for example, [15,24,27-31]).

Since functional networks have proven useful for identifying single genes functionally related to a gene set, we hypothesized that a global-network approach would also assist in identifying modules of genes functionally related to a gene set (Figure 1d). Here we present Reflective Diffusion and Local Extension (RIDDLE), an integrative method for systematically interrogating if a query gene set lies close to a known functional pathway in a genome-scale functional protein network. We show that the combinatory use of global-network, local-network, and classic enrichment information more reliably identifies relevant gene sets than existing methods. Notably, we can find functionally related sets even when the query gene set is sparsely or not at all annotated. Because RIDDLE can measure association between any two gene sets that are contained within the network, it is potentially applicable to a wide variety of settings without the need for additional pathway-specific information. As an example, a search for diseases associated with predicted microRNA (miRNA) targets led us to discover evidence of an ocular acting miRNA - a finding supported by literature and our own experimental confirmation of developmental mouse eye gene expression analyses. A web-based implementation of our method is available.

## Results and discussion

### Overview of RIDDLE methodology

**Figure 2:**
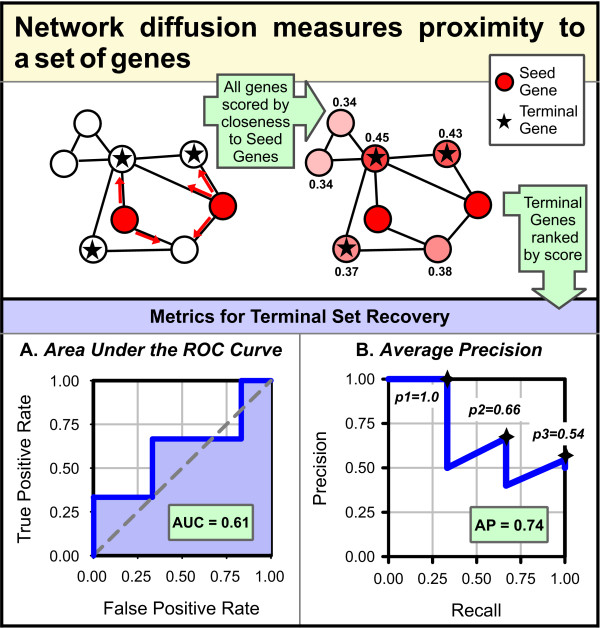
**Reflective diffusion (RD) operates on the principal that a seed set predicts a terminal set if the terminal genes are ranked highly by network diffusion score**. Recovery is measured by area under the ROC curve (AUC) or average precision (AP). Higher scores for both measures indicate stronger functional association.

RIDDLE contains two key independent parts, reflective diffusion (RD) and local extension (LE), and uses a human functional interaction network developed previously [15]. In principle, any other such genome-scale functional gene network could be employed (for example, [11,12,16,30,32,33]) provided they exhibit high coverage of the set of human genes and high specificity for the gene-gene interactions. Given a query set, RIDDLE examines the network for closely positioned known functional gene sets. For the RD component, we adapt a diffusion algorithm shown previously to work well with our network [15,30]. RD evaluates overall connectivity between a query set and known pathway set by loading one (seed set) into the diffusion algorithm [30], then assessing how well a second (terminal set) is predicted or recovered by the measures area under the ROC curve (AUC) and average precision (AP) (Figure 2). The diffusion analysis is repeated such that both the query and known pathway sets have a turn to serve as the seed set (thus the term reflective). Note that in this context, AUC and AP are employed as convenient summary statistics of the proximity of two gene sets within the network, regardless of whether the gene sets are indeed functionally associated.

**Figure 3:**
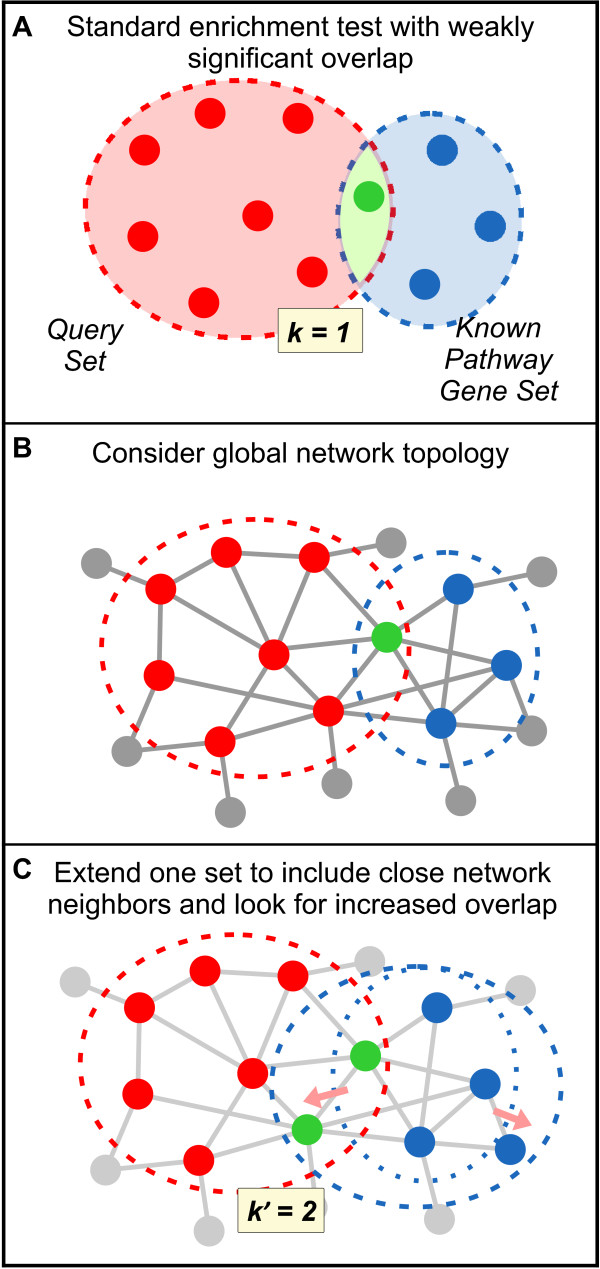
**Local extension (LE) adds functionally associated genes to increase the overlap between two gene sets**. **(a) **Local extension is particularly useful when a weakly significant overlap between a query gene set and a known functional set is detected by a classic enrichment method. **(b) **The positions of the genes in a global functional network are consulted. **(c) **Close network neighbors are added to one set, and the significance of the newly formed overlap is re-calculated.

Meanwhile, the LE component performs a more localized interrogation, extending either the query or pathway set to include strongly implicated direct neighbors [18] and checking for improved enrichment with each new set (Figure 3). In gene set enrichment analyses, a query set commonly overlaps weakly with multiple functional gene sets. The extension process serves to strengthen genuine functional relationships; spurious matches are less likely to be improved with network information.

Naturally, overlap-based methods perform most strongly when a substantial overlap exists between the query set and the relevant functional set. In contrast, network-based methods dominate when little or no overlap is present (as we will describe fully in the following sections). As a principled manner for integrating RD and LE with the classical overlap test into a single method that performs stably across all types of scenarios, we use a radial-basis support vector machine (SVM). Additionally, a machine learning approach is ideal for capturing any complex relationships that may exist between different measurements. Finally, we note that while powerful, network analyses must be used discerningly to avoid introducing systematic biases or artifacts, some of which we describe below.

### Centrality and size affect network-based measurements

In order to first survey general properties of functional gene sets in the network, we used RD-AUC to measure the connectivity between various combinations of pathway gene sets defined in the Kyoto Encyclopedia of Genes and Genomes (KEGG) and random gene sets. Surprisingly, resulting AUCs are not necessarily near 0.5, but seem to vary according to certain characteristics of the genes involved. To demonstrate the possible range of these intrinsic AUCs, we used 100 random seed sets of fixed size and weighted node degree to predict each KEGG set. The mean AUC for each KEGG set ranges from 0.164 to 0.849, and the standard deviation from 0.003 to 0.160 (Figure S1a in Additional file 1). Figure S1b in Additional file 1 displays in detail the distributions for a range of KEGG sets; some gene sets, such as genes for glycerophospholipid metabolism, are clearly more difficult to recover, while others, such as genes annotated for renal cell carcinoma, are easier to recover.

**Figure 4:**
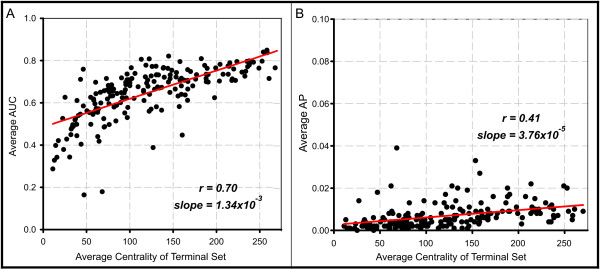
**Central gene sets tend to score higher AUCs and APs**. **(a) **In tests using random seed sets of various sizes and centrality to predict KEGG sets, the centrality of the terminal set correlates strongly with AUC (*r *= 0.70, slope = 1.34e-3). **(b) **Centrality of the terminal set also correlates with AP but to a lesser degree (*r *= 0.41, slope = 3.76e-5).

We found the strongest predictor for intrinsic AUC to be the average centrality, or sum of connecting network edge weights to a gene, of the members of the terminal set. As shown in Figure 4a, the average centrality of the terminal set is positively correlated with the average AUC obtained from random seed sets of a fixed size and centrality (*r *= 0.70, slope = 1.34e-3). This confounding relationship between AUC and centrality has recently been described and examined extensively [34]. Intuitively, a well-connected gene in the network interacts with more partners and is more likely to be involved in any given function. However, we cannot easily distinguish between a biological hub and a well-studied gene. Moreover, centrality should certainly not overweigh the other pieces of information that factor into a prediction algorithm. However, by accounting for this behavior in some fashion - for example, by considering a relative AUC score - AUC can, in principle, still be used to make genuine predictions. Importantly, in the next section we report that AUC may indeed be successfully employed as a measure of gene set connectivity and, furthermore, ranking gene sets by highest average centrality can only achieve a small fraction of this performance (Figure S3 in Additional file 1).

**Figure 5:**
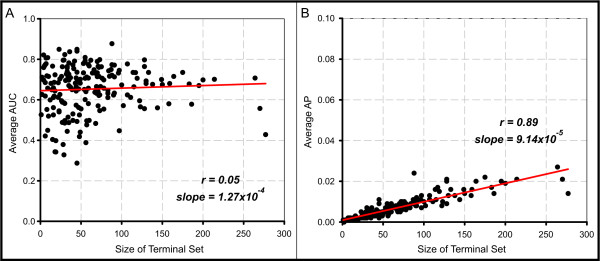
**Larger gene sets tend to score higher APs**. **(a) **In tests using random seed sets of various sizes and centrality to predict KEGG sets, the size of the terminal set is not correlated with AUC (*r *= 0.05, slope = 1.27e-4), but **(b) **correlates strongly with AP (*r *= 0.89, slope = 9.14e-5).

We also looked for similar unexpected trends when using RD-AP to measure connectivity between random and KEGG gene sets. Here, the average scores obtained for predicting KEGG sets range from 0 to 0.039 (Figure S2 in Additional file 1). Though AP in principle ranges from 0 to 1, the random APs are more tightly distributed around a low value (0.006). AP is also affected by terminal set centrality; however, the slope of the relationship is much less steep (Figure 4b; *r *= 0.41, slope = 3.76e-5). We found that terminal set size is a much stronger predictor of AP but not AUC (Figure 5; *r *= 0.89, slope = 9.14e-5). To help understand why AP would tend to increase with terminal set size, consider the extreme case where the terminal set equals the entire set of known genes. Because every gene is contained within the terminal set, the AP will equal 1. Notably, if we normalize AP by the terminal set size, the correlative trends with size and centrality diminish greatly (Figure S6 in Additional file 1). Finally, we looked for trends associated with seed set characteristics, but these were relatively minor (data not shown).

These results suggest that network-based analyses and, more generally, performance measures should be carefully evaluated. Interestingly, when measuring the same data set, centrality more strongly affects AUC than AP, while size affects AP but has no apparent influence on AUC. In the apparent lack of a perfect performance measure, we find a productive solution is to apply great care in properly interpreting a measure for the task in question. For example, while many pathways will sit close to a highly central gene set, a match is only interesting if it is exceptionally strong. We show below that AUC and AP are clearly effective for correctly identifying associated gene sets. Because of these unexpected trends we observed with AUC and AP, we chose to employ an SVM to model any complexities that exist between our performance measures and centrality, size, and other relevant gene set features.

### Application to simulated data sets

Given these observations on the intrinsic predictability of functional gene sets using the network, we next wished to assess the utility of RIDDLE for correctly associating functionally related gene sets. We therefore created subsets of known gene sets and tested our method's ability to correctly match the subsets. Specifically, we created several types of tests from genes in KEGG and Gene Ontology (GO) pathways, where each test case consists of a pathway divided into a query subset and a known subset. For each query subset, we ranked all generated known subsets by their RIDDLE association score (RAS). We purposefully designed tests where subsets of a pathway are excluded from sharing genes, simulating the extreme case where query genes are completely unannotated. Also, we note that KEGG information was not incorporated into constructing the functional network, reducing the potential for logical circularity. As further affirmation of the independence between GO and KEGG, the Jaccard similarity coefficient between linkages defined by the two databases is 0.017, or only 6.4% of all KEGG-based links.

**Figure 6:**
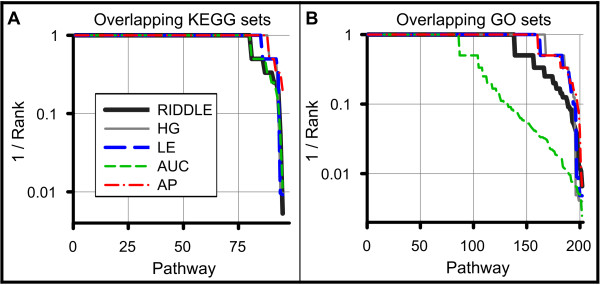
**Most methods perform comparably in matching overlapping gene subsets**. **(a,b) **RIDDLE matches most overlapping subsets of various gene sets created from KEGG pathway sets (a) and GO biological process sets (b). The reciprocal of the rank of the matching subset is shown for RIDDLE, the RD and LE components in the reverse direction, and the hypergeometric test (HG).

We first assessed test cases allowing overlap, which were created by random drawings from known gene sets with replacement. Here, RIDDLE correctly recovers matching subsets for 84% and 68% of KEGG and GO gene sets, respectively (Figure 6a,b), nearly matching the performance achieved by a hypergeometric test. The performance is robust to the gene set database used, for the trend is similar across subsets created from KEGG and GO.

**Figure 7:**
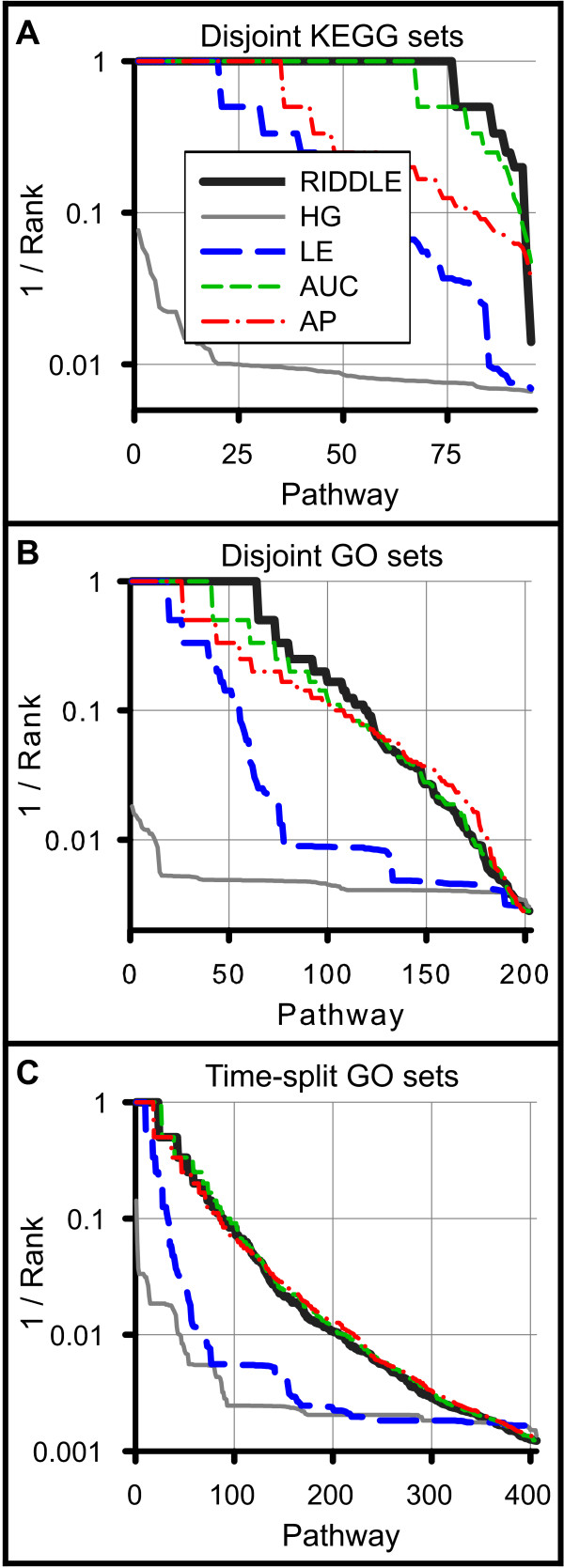
**RIDDLE performs superiorly in matching disjoint gene subsets**. **(a-c) **RIDDLE matches many disjoint subsets of various gene sets created by dividing KEGG pathway sets (a) and GO biological process sets (b) randomly into non-overlapping halves, and splitting GO biological process sets (c) by those annotated prior to and after 9 February 2007. Note the failure of hypergeometric for all three cases and the incremental improvement obtained by each component and the final combinatory RIDDLE method.

Next, we considered test cases explicitly containing no overlap: (1) KEGG and (2) GO sets split disjointly in half and (3) GO genes present before and added after 5 February 2007. Here, not surprisingly, the hypergeometric test fails catastrophically and does not perfectly match any subsets (Figure 7a-c). In these tests, the benefit of the individual components of RIDDLE is clear; a substantial number of matches are recovered utilizing LE alone, and even more so with RD. Overall, RIDDLE correctly matches pathways for 80% of KEGG test cases. GO cases present a significantly harder challenge for all methods; 31% and 5% of GO random-split and time-split cases are recovered by RIDDLE. Though GO time-split cases are the most difficult, a clear advantage is gained by employing network diffusion.

The hypergeometric test and the individual RIDDLE components each exhibit individual areas of extreme strength and weakness (summarized in Table 1; detailed results of each component are shown in Figure S3 in Additional file 1). However, the combined RIDDLE method is stable across all test types; RIDDLE nearly matches or bests the other components regardless of the test database or the allowance or exclusion of overlap between subsets. We also note that many gene sets within KEGG and GO are closely related, adding another level of difficulty to these tests. Highly ranked pathways, while not the correct match, are often biologically related. For example, RIDDLE identifies 'axon guidance' as the top match for 'neuron projection development', 'triglyceride metabolic process' is matched with 'steroid and cholesterol metabolic process', and 'response to virus' is matched with 'innate immune response', 'response to bacterium', and 'defense response to virus'. In fact, RIDDLE can correctly match many sibling gene sets of the same category in the KEGG hierarchy (Figure S4 and Table S1 in Additional file 1).

**Table 1:** Strengths and weaknesses of the hypergeometric test and individual components of RIDDLE

Method	Strengths	Weaknesses
Hypergeometric	Gold standard for overlapping sets	Fails for split sets

Local extension	Moderate improvement upon split sets	

Diffusion - AUC	Exceptional improvement upon split sets	Considerable performance loss for GO overlap sets Strong correlation with centrality

Diffusion - AP	Matches hypergeometric for overlapping sets Major improvement upon split sets	Weak correlation with centrality Strong correlation with set size

RIDDLE	Best for split sets	Minor performance loss for GO overlap sets

AP, average precision; AUC, area under the ROC curve; GO, Gene Ontology.

### Matching microRNA targets with disease genes

Finally, we looked for functional associations of miRNAs. miRNAs are thought to regulate gene expression by repression, but due to widespread gene targeting, the overall functionality of particular miRNAs is poorly understood. We matched predicted targets of miRNAs with disease-associated genes (Table S2 in Additional file 2). Generally, most miRNA targets seem to be associated with a large number of diseases, agreeing with a growing body of evidence linking individual miRNAs to multiple biological pathways and diseases [35]. However, we discovered an interesting case of a miRNA that scores highly with numerous eye diseases.

**Figure 8:**
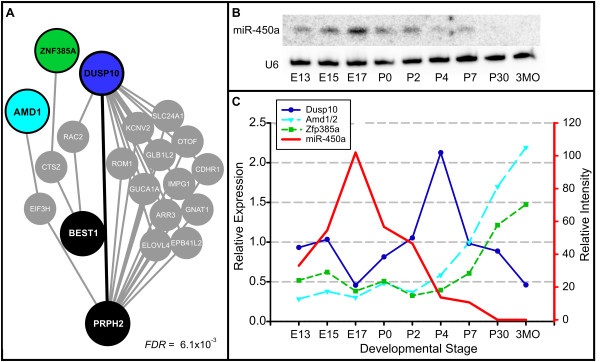
**Predicted targets of miR-450a and ocular disease genes are disjoint, yet functionally associated**. **(a) **Targets of miR-450a (*DUSP10*, *AMD1*, *ZNF385A*, colored nodes) and genes associated with macular dystrophy (*ELOVL4*, *BEST1*, *PRPH2*, black nodes) are 'close' to each other in the human functional network (false discovery rate (FDR) ≤8.54e-3). There are no shared genes and direct connections are indicated by bold edges. **(b) **Northern analysis of mouse eye total RNA suggests miR-450a is active in eye development during embryonic stages. U6 spliceosomal RNA serves as a loading control. **(c) **In contrast to miR-450a, predicted targets Dusp10, Amd1, and Zfp385a (mouse homolog of ZNF385A) are expressed at low levels during early stages.

Three predicted targets of miR-450a - Dusp10, Amd1/2, and Znf385a - are significantly close to genes of numerous different eye-specific diseases in the network. Four of the top six disease matches for miR-450a are eye-specific (Table 2). Remarkably, none of the disease genes, which are mostly unique across the diseases, are shared with the miRNA targets. For example, miR-450a targets are completely disjoint with macular dystrophy genes and share only a single direct connection (Figure 8a). The homologue of miR-450a is already known to be expressed in the cornea in mouse [36]. We performed an additional Northern analysis on total RNA from mouse eyes across various stages of development and found that, consistent with a role in the developing eye, miR-450a is expressed from embryonic day 13 (E13) to postnatal day 7 (P7), peaking near E17, and undetectable in adult eyes (Figure 8b). Quantitative real-time PCR experiments show expression of miR-450a's predicted targets to be lower during these stages (Figure 8c). Dusp10 expression increases after P2 but falls again after P4, suggesting the presence of additional gene regulation. Overall, these data confirm miR-450a expression in the mammalian eye, suggest that miR-450a plays a regulatory role in eye development and supports the predicted linkage of the miRNA to eye diseases.

**Table 2:** Top ten OMIM diseases functionally associated with predicted targets of miR-450a

	OMIM ID	Description	RAS	FDR
*1	608161	Macular dystrophy, vitelliform, adult-onset	0.08838	0.006
*2	136880	Fundus albipunctatus	0.08833	0.006
3	212750	Celiac disease	0.08820	0.010
4	123400	Creutzfeldt-Jakob disease	0.08819	0.010
*5	258100	Oguchi disease	0.08818	0.010
*6	248200	Stargardt disease	0.08815	0.012
7	158350	Cowden disease	0.08811	0.016
8	612242	Chromosome 10q23 deletion syndrome	0.08811	0.016
9	185800	Symphalangism	0.08811	0.017
10	254780	Myoclonic epilepsy of lafora	0.08810	0.019

Starred entries indicate eye-specific diseases. FDR, false discovery rate; OMIM, Online Mendelian Inheritance in Man; RAS, RIDDLE association score.

Arriving at a tissue-specific role for a miRNA without the use of any tissue-specific data was a surprising and interesting result. While some large-scale tissue-specific gene expression data are available via *in situ *experiments (for example, [37]), pigment color further hinders eye-specific expression measurements. RIDDLE discovered this functional association without any knowledge of the expression patterns of miR-450a or the predicted targets. We also note that this association relied entirely on RIDDLE's use of network connectivity, as no genes were shared between the miR-450a targets and the disease pathways (Figure 8a).

## Conclusions

We tested our method across the two possible extremes when identifying functional enrichment: either a substantial amount or none of the query set is annotated. We see that RIDDLE is best for the latter extreme without compromising much performance in the former. In contrast, the standard hypergeometric test - or any other method relying on the assumption of well-annotated query sets - fails catastrophically for split cases. Unfortunately, knowing where data lie in this spectrum of 'overlap' is only possible in simulations. Thus, RIDDLE serves as a robust, general purpose method, drawing on the strengths of each individual component. We note that it nonetheless has intrinsic limitations, most notably, that it is limited by the gene coverage of the network (currently 87% of the human genes [15]).

Many sophisticated schemes exist for finding functional association, encompassing a wide range of data transformations and statistical models (discussed extensively in [38]). Regardless of the approach used, the success of an analysis requires that the query and pathway genes are well-annotated. While approximately 78% of protein-coding genes currently have some level of annotation in the commonly used GO database, a substantial portion of these genes have only minimal, high-level annotations. There are many local network-based methods, though in addition to adequate annotations, they require a detailed mapping of the pathway interactions, of which only a limited number are available. Thus, a global network-based method provides a feasible alternative when only partial information is known of the query or pathway genes.

Recently, a number of global network-based methods have been developed, an indicator of the progression and growing importance of this strategy. For example, GsNetCom, a method using cumulative shortest path length can correctly match most overlapping sets and, indeed, many disjoint sets [39]. However, the method falls short of RIDDLE for disjoint sets, once again asserting the benefit of considering whole network topology through a diffusion algorithm, and furthermore, the power of using an integrative method (Figures S3 and S5 in Additional file 1). RIDDLE does benefit from using a more complete network with edges assembled from quality-weighted data, for implementing the GsNetCom method with HumanNet obtained a moderate improvement (Figure S5 in Additional file 1). In another example, Huttenhower and colleagues [16] define the association between gene sets as the amount of cross-talk, or strength of direct linkages between two gene sets (see also the similarly based method by Li *et al. *[10]). This slightly less robust method performs comparably with RIDDLE in many but not all test cases (Figure S5 in Additional file 1). Additionally, other global network applications have emerged for the highly related but distinct task of analyzing gene expression microarrays (for example, [40]). These methods have demonstrated the utility of mapping differential expression onto a protein interaction network for deducing pathway level changes [41-43]. Again, all of these different methods highlight the utility of using global network information in various analyses.

In the past decade, we have seen the extensive development of gene networks and their accompanying functional gene analyses (reviewed in [24,31]). With the continual production of genome-scale data, network-based analyses are likely to become even more necessary. Here, we have established a means for utilizing information-rich networks to understand gene function, achieved through a few adaptations to existing GBA methods. There are many previously established network-based methods among which RIDDLE performs competitively, if not better. We have demonstrated multiple instances where the method is uniquely useful, such as applying RIDDLE to link a miRNA to an array of likely relevant diseases, even when none of the gene sets overlap. RIDDLE potentially benefits a wide range of applications that may require the functional characterization of poorly understood gene sets.

## Materials and methods

### Functional network

We use the human functional interaction network described in [15]. This network contains 476,399 links among 16,243 genes (87% of protein coding genes) and is constructed from various distinct lines of evidence.

### Data sets

Pathway genes were downloaded from KEGG [44,45] on 23 April 2010. GO sets were downloaded [46,47] on 7 February 2007 and 17 April 2010. In total, we acquired 811 GO biological process terms with highly reliable evidence (IDA, IEP, IGI, IMP, IPI, and TAS). Conserved targets of 153 human miRNA families were predicted by Targetscan [48] and downloaded [49] on 15 June 2011. Genes associated with human diseases were obtained from the Online Mendelian Inheritance in Man (OMIM) [50] on 24 August 2008. In total, 497 multi-gene sets are included in our match algorithm.

### Simulated data sets

We created various subsets of KEGG and GO gene sets. For a pathway of size *n *genes, we created the following subsets: (1) two independent draws of 0.5 × *n *genes from the pathway (allowing overlaps) and (2) a random division of the pathway into two approximately equal sized, non-overlapping sets. For GO gene sets, we created an additional set of divisions by annotation time: genes known in the 2007 version and genes unique to the 2010 version. In total, we compiled the following data sets: 190 of each overlapping and disjoint KEGG sets, 404 of each overlapping and disjoint GO sets, and 811 time-split GO sets. To create random sets used for determining AUC and AP correlation with centrality and size, we selected a KEGG set with average centrality approximately 100, matching the mean value among all KEGG sets (with outlying sets of average centrality >200 removed). In order to hold size and centrality constant over randomization, network genes were first split into 10 equally spaced bins by centrality, then for each of the genes in the set, we randomly drew a gene from the corresponding bin.

### Hypergeometric test

To test if a query set significantly overlaps with a pathway set, we obtained the following *P*-value:

$$p\left(x\geq k\right)=\overset{\text{min}\left(n,m\right)}{\underset{x=k}{\sum }}\frac{\left(\begin{array}{c} m\\ x \end{array}\right)\left(\begin{array}{c} N-m\\ n-x \end{array}\right)}{\left(\begin{array}{c} N\\ n \end{array}\right)}$$

where *N *is the number of known genes, *m *is the number of genes in the pathway, *n *is the number of genes in the query set, and *k *is the size of the overlap.

### Local extension

To measure the local connectivity between two gene sets in the network, *s1 *and *s2*, we extend *s1 *to include nearby neighbors in the functional gene network, with 'nearness' of a particular gene determined by the sum of connecting edge weights to the gene set. This is followed by a hypergeometric test to measure the significance of the overlap between the extended set *s1' *and *s2*. To avoid over-extending the pathway to include non-specific pathway associations, we implemented the following cutoff rule:

$$\text{LE}\left(\text{n}\right)=\text{min}\left(\alpha \cdots \text{n,}\;\beta \right),$$

where the maximum size of the extension for the pathway with *n *genes depends on the two free parameters *α*, the percentage of the size of *s1*, and *β*, the maximum size of extension. This ensures that the degree of extension is proportional to the original size of *s1*. If necessary, we allow the maximum size to be breached in order to accommodate multiple genes of equal score. We found LE to perform optimally with α and β equal to 0.8 and 100, respectively.

### Reflective diffusion

To measure connectivity between two gene sets in the network, *s1 *and *s2*, we adapted the diffusion algorithm described in [15,30] (Figure 3). Briefly, given *s1 *as the input seed set, the algorithm ranks all other genes in the network by how strongly connected they are to the seed set. We then have two means of measuring how well this ranked list recovers *s2*, the terminal set: (1) AUC and (2) AP.

To calculate AUC, we plot true-positive rate (TP/(TP + FN)) as a function of the false-positive rate (FP/(FP + TN)), then find the corresponding AUC. A higher area indicates better recovery. To calculate AP, we sort the *k *genes of *s2 *by rank and then average the precision, or fraction of the set recovered, achieved for each member of the set:

$$AP=\frac{1}{k}\overset{k}{\underset{i=1}{\sum }}\frac{i}{rank_{i}}.$$

### RIDDLE - combining RD and LE

To measure the connectivity between a query set and a pathway set, we perform the following: a hypergeometric test, forward direction tests (LE and RD with query and pathway sets as *s1 *and *s2*, respectively), and reverse direction tests (LE and RD with pathway and query sets as *s1 *and *s2*, respectively).

To combine the results, we used libsvm, a library of SVM software implemented in C with a Matlab interface [51] downloaded from [52]. We chose a radial basis kernel trained with the following features: log *P*-values from the hypergeometric, forward LE, and reverse LE tests, forward RD-AUC, reverse RD-AUC, forward RD-AP, reverse RD-AP, query size, pathway size, overlap size, query set average centrality, pathway set average centrality, and percent of query genes contained in the network. As positive training data, we used the simulated KEGG and GO split sets. As negative training data, we included ten mismatched pairs per simulated query set, plus random sets of varying size paired with a randomly chosen real (KEGG or GO) set (167 sets total).

To determine a good combination of kernel parameters to use, we used a cross-validation and grid search technique. We divided the aggregate training data into modeling (25%), cross-validation (25%), and final validation (50%) sets. Overall, for modeling, we used 498 positive pairs and 5,147 negative pairs. Because we had many more examples of negative matches, we used a lower weight cost for the negative class. For each combination of parameters we trained with modeling data and measured performance with cross-validation data. We chose a final model based on strong performance with both overlapping and split data types and report the performance for the final validation set. The final parameters for our SVM are: positive match class weight *w1 *= 1, negative match class weight *w0 *= 0.3, cost *C *= 10e8, termination criterion *e *= 0.01, kernel parameter *γ *= 0.07.

The RAS is the score output from the trained SVM. We calculated an empirical false discovery rate using final validation matched subsets to generate a positive RAS distribution and random gene sets paired with KEGG and GO sets to generate a negative RAS distribution (Figure S7 in Additional file 1). For calculating the false discovery rate, we normalized both positive and negative distributions to have a total area of 1, though in principle, the likelihood of a negative match is much greater.

### GsNetCom

Synthetic data sets were input into the batch tool gene similarity calculator available online [39,53]. Known sets were ranked by the resulting corrected cumulative rank score (CCRS). Additionally, the algorithm as described by Wang and colleagues [39] was implemented with HumanNet.

### Crosstalk

The algorithm for 'Functional mapping associations' as described by Huttenhower and colleagues [16] was implemented with HumanNet weighted linkages.

### Gene expression analysis

Mouse eye total RNA samples were obtained from Zyagen (San Diego, CA, USA). Three mice were dissected for each of embryonic stages E13, E15, and E17, two mice were used for each of postnatal stages P0, P2, P4, and P7, and 1 mouse was used for each P30 and 3 month stages.

Small RNA Northern blot analysis was performed as previously described [54,55]. Briefly, 10 μg of total RNA was separated on a Tris-borate-EDTA-urea-15% polyacrylamide gel. The RNA was then transferred to a Hybond N+ membrane (GE Healthcare, Waukesha, WI, USA), UV cross-linked, and pre-hybed for 1 hour in ExpressHyb buffer (Clonetech, Mountain View, CA, USA) at 55°C. Oligonucleotide probes (Integrated DNA Technologies, Coralville, IA, USA) were radiolabeled using [gamma-32P]ATP (Perkin Elmer, Waltham, MA, USA) and T4 polynucleotide kinase (New England Biosciences, Ipswich, MA, USA). Labeled probes were hybridized overnight at 38.5°C followed by four washes with 2× SSC, 0.1% SDS solution. Storage phosphor screens (GE Healthcare) were exposed and scanned using a Personal Molecular Imager system (Biorad, Hercules, CA, USA). Blots were stripped by washing with boiling 0.1% SDS. Probe sequences used were: U6, CGTTCCAATTTTAGTATATGTGCTGCC; miR-450a, ATATTAGGAACACATCGCAAAA.

Total RNA from each stage was reverse transcribed using Superscript II reverse transcriptase (Invitrogen, Grand Island, NY, USA) and random hexamers. For each sample and target, gene expression was measured in triplicate with an ABI ViiA 7 real-time PCR system using SYBR Green (Invitrogen). We constructed standard curves and measured efficiency for each probe using known dilutions of pooled cDNA composed of each stage. For each sample, gene expression was calculated from median values and normalized to the expression of reference gene 18s ribosomal RNA. Probe sequences used are (forward and reverse): 18srRNA, AGTGCGGGCCATAAGCTTGCGT, GCCGTGGGCCTCACTAAACCATCCA; Dusp10, TCGAGGAAGCTCACCAGTGTGGGA, TAGGCGATGACGATGGTGGCGGAT; Amd1/10, GTCTCACGGTGATGGAAGCTGCAC, TCCCTGGCTTGCGTCGGACT; Zfp385a, AGGGAGCCTAGTGTCCGGGAATCA, TGGAAACTGGACGAGGGGCTACAC.

## Abbreviations

AP: average precision; AUC: area under the ROC curve; E: embryonic day; GBA: guilt-by-association; GO: Gene Ontology; KEGG: Kyoto Encyclopedia of Genes and Genomes; LE: local extension; miRNA: microRNA; P: postnatal day; RAS: RIDDLE association score; RD: reflective diffusion; RIDDLE: Reflective Diffusion and Local Extension; SVM: support vector machine.

## Competing interests

The authors declare that they have no competing interests.

## Authors' contributions

PIW and SH carried out the study design and data analysis. RPK and CSS designed and carried out Northern blot experiments. IL and EMM conceived and supervised the study. PIW drafted the manuscript. All authors read, revised, and approved the final manuscript.

## Supplementary Material

Additional file 1 **Supplementary Figures S1 to S7 and Table S1**. Click here for file

Additional file 2 **Supplementary Table S2**. Results produced using RIDDLE to match predicted targets of miR-450a with OMIM disease genes. Click here for file
