# Supplementary material for: RIDDLE: reflective diffusion and local extension reveal functional associations for unannotated gene sets via proximity in a gene network
Source: Genome Biol. 2012 Dec 26;13(12):R125. doi: 10.1186/gb-2012-13-12-r125 (PMC4056375; doi:10.1186/gb-2012-13-12-r125)
Supplement: Additional file 1 — Supplementary Figures S1 to S7 and Table S1. [file gb-2012-13-12-r125-S1.DOCX]

**Additional File 1**

**Supplemental figures for the article:**

**RIDDLE: Reflective diffusion and local extension reveal functional associations for unannotated gene sets *via* proximity in a gene network**

Peggy I Wang^1,2,6,†^, Sohyun Hwang^3, †^, Rodney P. Kincaid^1,4,6^, Christopher S. Sullivan^1,4,6^, Insuk Lee^3,*^, and Edward M Marcotte^1,5,6,*^

**
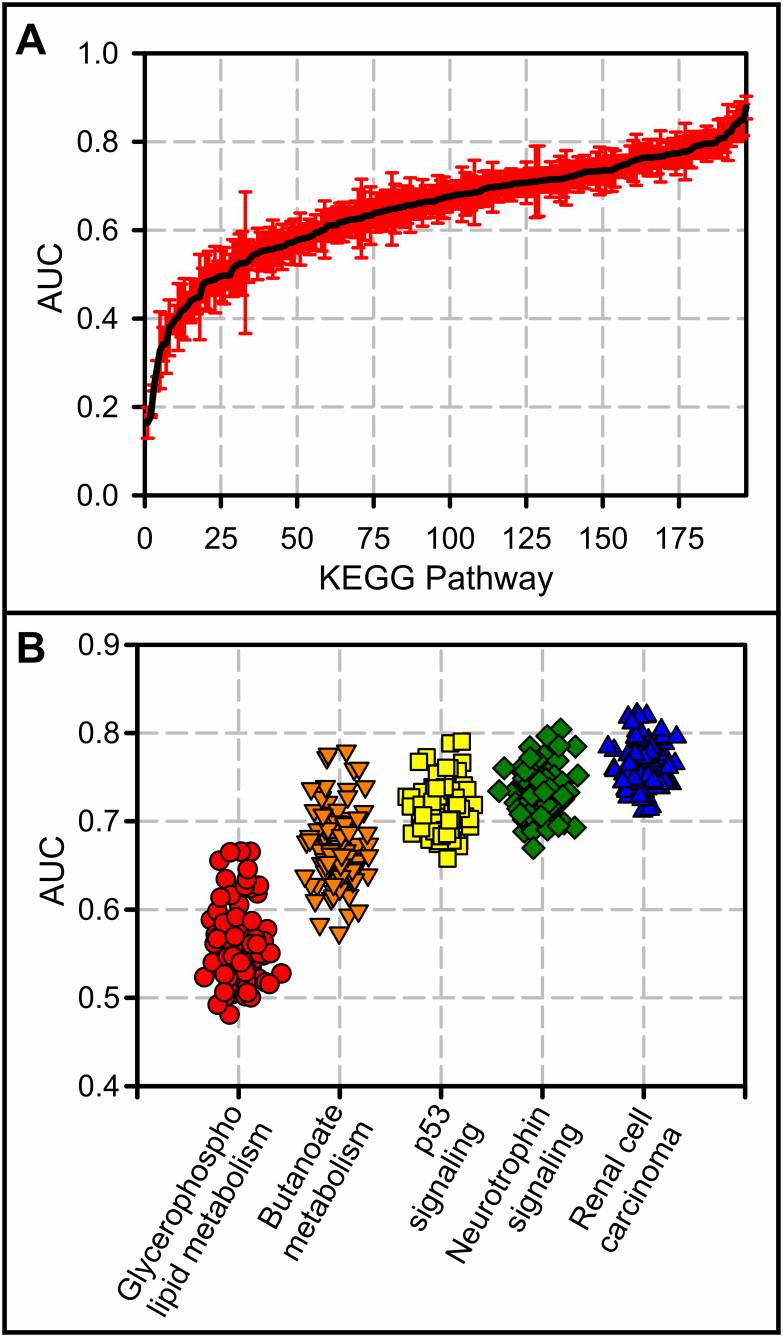
**

**Supplemental Figure S1.** Each KEGG pathway set has an intrinsic predictability, as measured by AUC. (A) For each KEGG set, the average and standard deviation of AUCs obtained using 100 random seed sets of fixed size centrality. (B) AUC distributions obtained for example pathways.

**
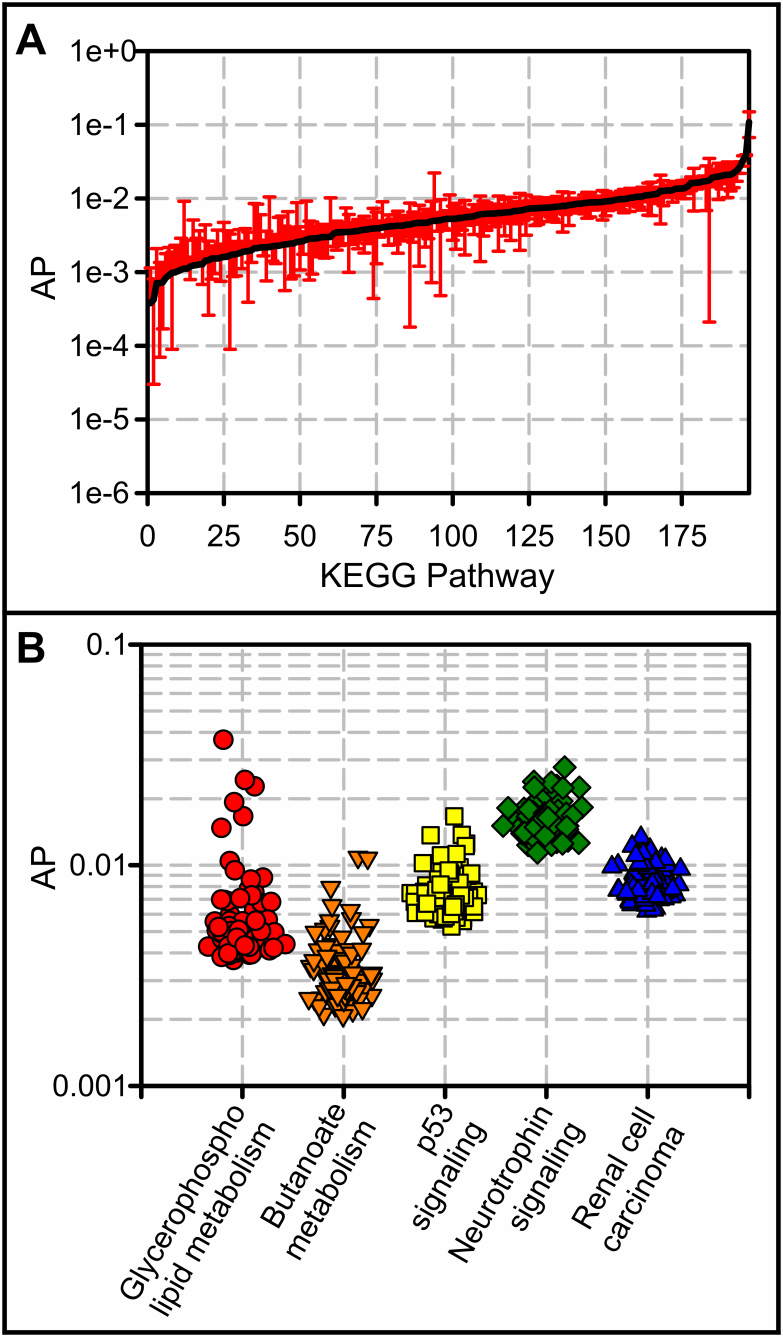
**

**Supplemental Figure S2.** Each KEGG pathway set has an intrinsic predictability, as measured by AP. (A) For each KEGG set, the average and standard deviation of AUCs obtained using 100 random seed sets of size and centrality. (B) AP distributions obtained for example pathways.


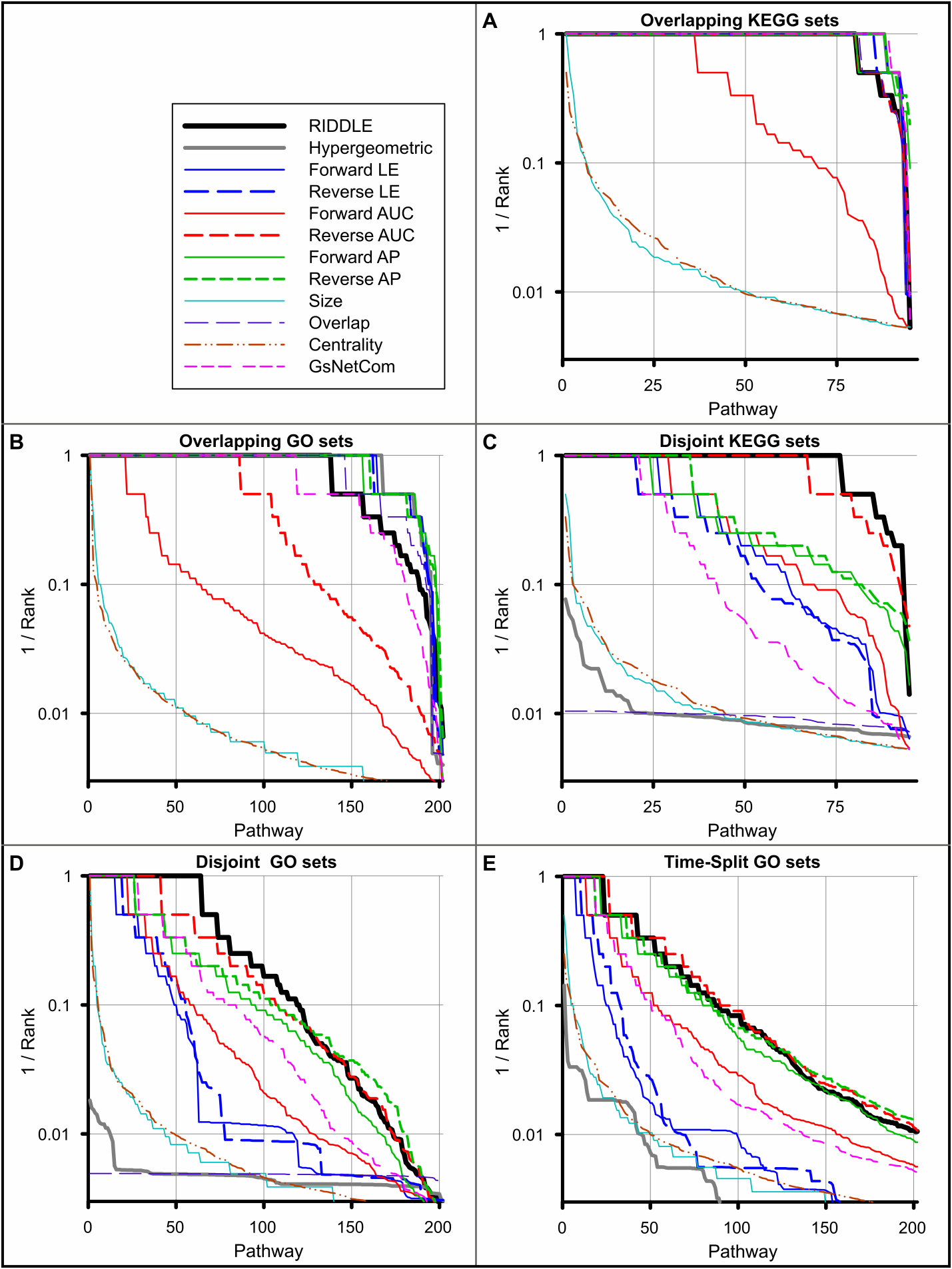


**Supplemental Figure S3.** Performance on matching simulated test sets using RIDDLE, components of RIDDLE (forward and reverse LE, RD-AUC, RD-AP, and HG), known set size, overlap, and known set average centrality, and previously established global-network method, GsNetCom. GsNetCom performs well for overlapping test cases (A,B). Though GsNetCom is among the best for overlapping KEGG sets, it performs worse than RIDDLE and components for disjoint KEGG sets (C). For disjoint and time-split GO sets, the method is bested by RIDDLE and diffusion-based components (D,E).

| **KEGG ID** | **Pathway description** | **HG** | **RIDDLE** | **GsNetCom  on HumanNet** | **GsNetCom** |
| --- | --- | --- | --- | --- | --- |
| hsa00130 | Ubiquinone and other terpenoid-quinone biosynthesis | 0.500 | *0.977* | 0.632 | 0.201 |
| hsa00670 | One carbon pool by folate | 0.558 | *0.955* | 0.542 | 0.221 |
| hsa00730 | Thiamine metabolism | 0.558 | *0.927* | 0.408 | 0.273 |
| hsa00750 | Vitamin B6 metabolism | 0.500 | *0.973* | 0.686 | 0.239 |
| hsa00770 | Pantothenate and CoA biosynthesis | 0.500 | *0.973* | 0.609 | 0.201 |
| hsa00780 | Biotin metabolism | 0.500 | *0.977* | 0.605 | 0.246 |
| hsa00785 | Lipoic acid metabolism | 0.500 | *0.967* | 0.658 | 0.351 |
| hsa00830 | Retinol metabolism | 0.553 | *0.943* | 0.587 | 0.198 |
|  | **Mean AUC** | **0.521** | ***0.961*** | **0.591** | **0.241** |

**Supplementary Table S1.** AUC results of the Hypergeometric test, RIDDLE, and GsNetCom (with the original published network and with HumanNet), for predicting “sibling” gene sets that belong to the KEGG category ‘Metabolism of cofactors and vitamins’.

**
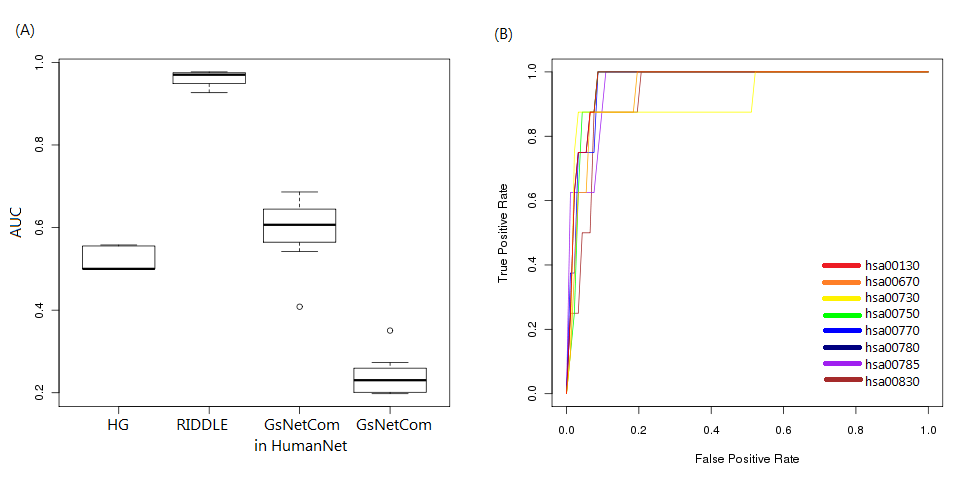
 Supplementary Figure S4.** AUC results of the Hypergeometric test, RIDDLE, and GsNetCom (with the original published network and with HumanNet), for predicting “sibling” gene sets that belong to the KEGG category ‘Metabolism of cofactors and vitamins’. (A) Each Box-and-whisker plot shows the distribution of AUCs by eight sibling gene sets for each method. (B) ROC curves by RIDDLE for eight sibling gene sets.

**
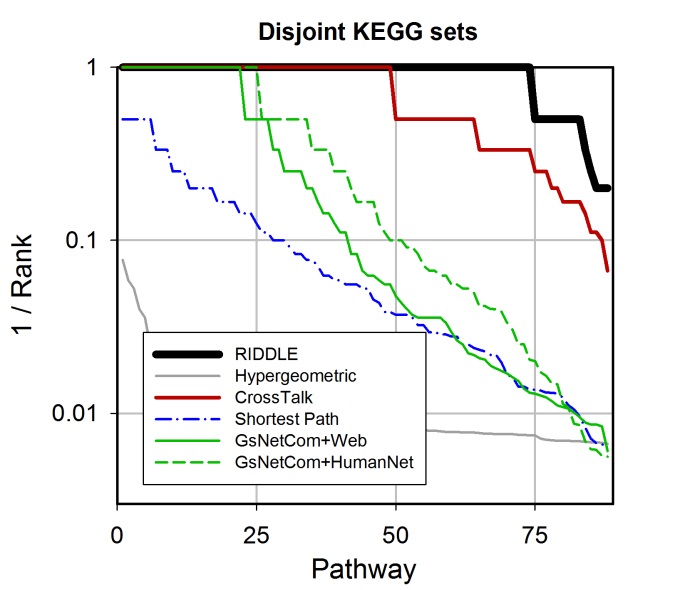

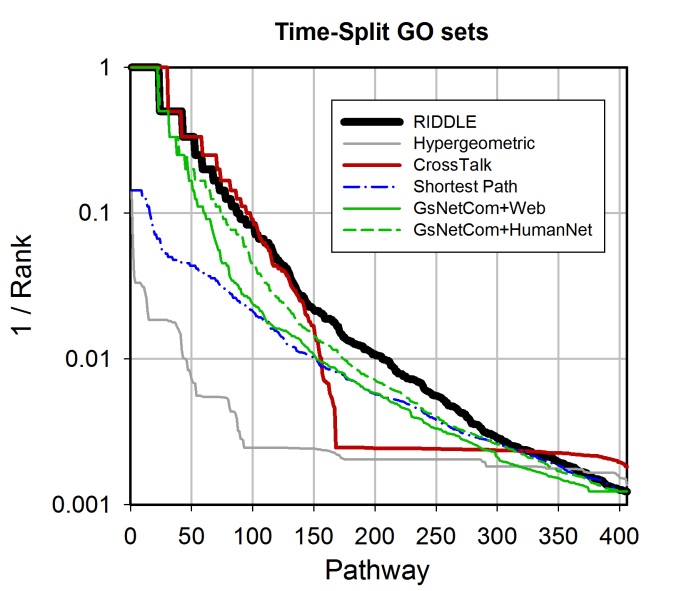
**

**Supplementary Figure S5.** Performance on matching disjoint sets using RIDDLE, the Hypergeometric test, Crosstalk (Li et al, Bioinformatics, 2008 and Huttenhower et al, Genome Research, 2009), and GsNetCom from the original web tool (Wang et al, Bioinformatics, 2011) and also implemented with HumanNet. Results for matching disjoint KEGG sets and time-split GO sets are shown on the left and right, respectively.

**Supplementary Figure S6.**

**
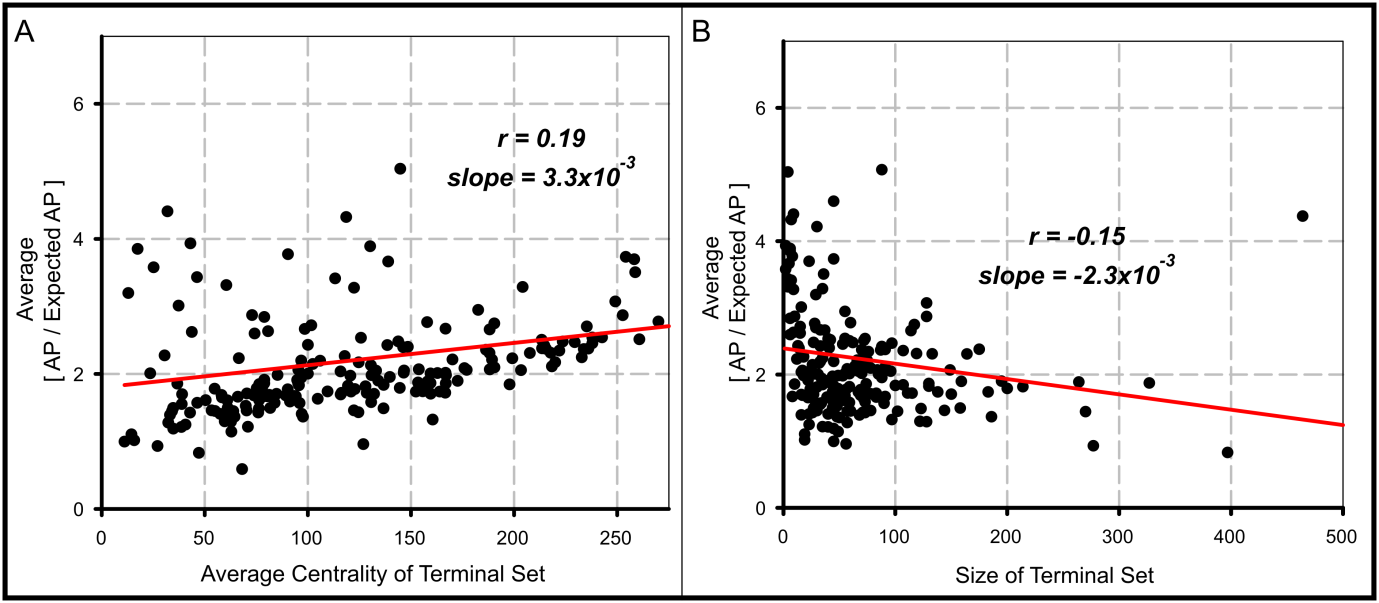
**

**Supplemental Figure S6.** AP values, when normalized by expected values, show weak correlation with centrality and size. Using random gene sets to predict known KEGG pathways, we measured the fold difference in AP over expected AP (number of genes in the KEGG pathway divided by the number of known genes). Though the correlation coefficients are very low for both set centrality (A) and set size (B).


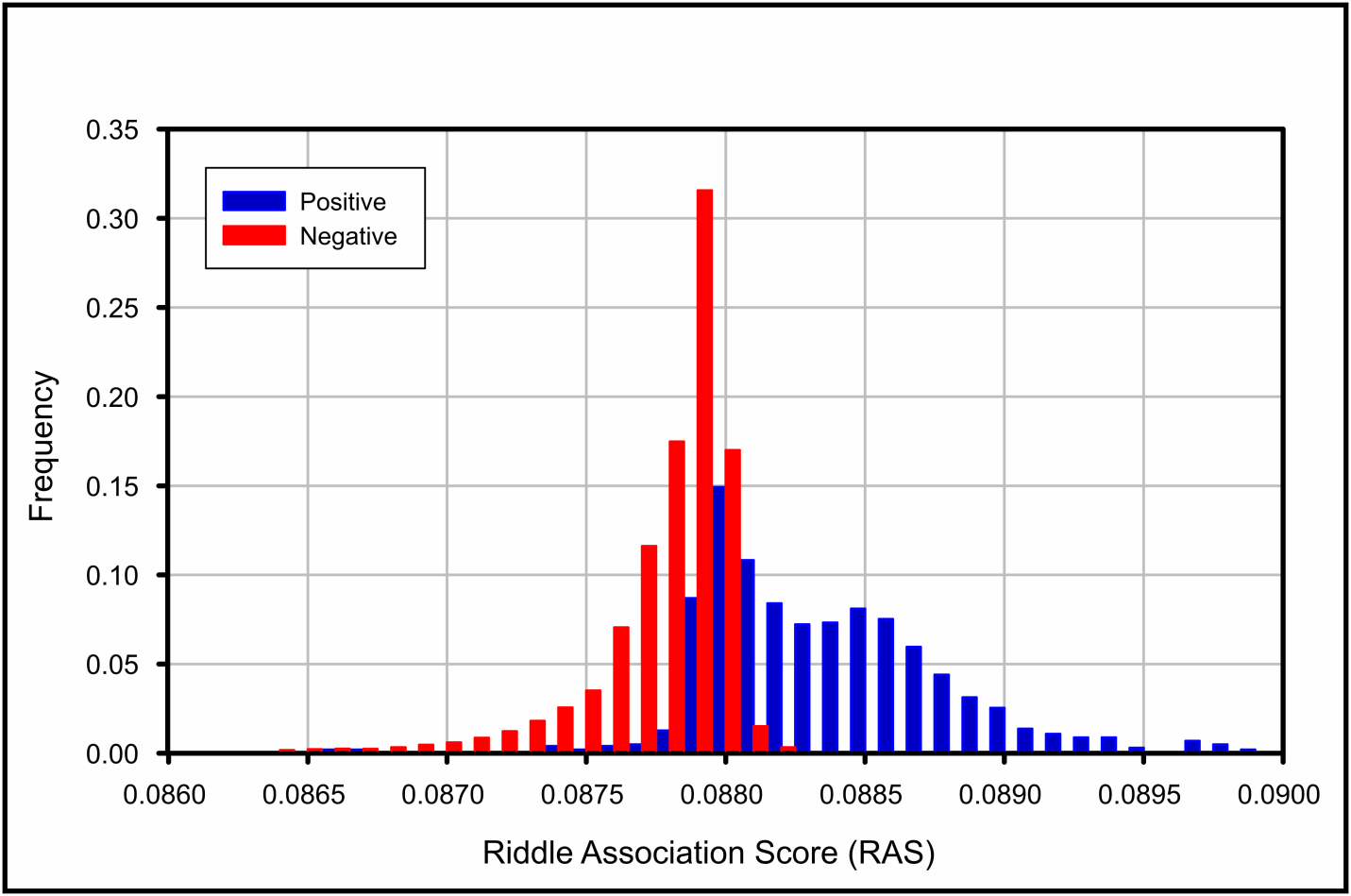


**Supplemental Figure S7.** Positive and negative RAS distributions for calculating an empirical FDR. Final validation matched subsets were used to generate a positive RAS distribution and random gene sets paired with KEGG and GO sets were used to generate a negative RAS distribution. Both positive and negative distributions are normalized to have a total area of 1.
